# Impact of hand hygiene intervention: a comparative study in health care facilities in Dodoma region, Tanzania using WHO methodology

**DOI:** 10.1186/s13756-020-00743-4

**Published:** 2020-06-08

**Authors:** Karin Wiedenmayer, Vicky-Sidney Msamba, Fiona Chilunda, James Charles Kiologwe, Jeremiah Seni

**Affiliations:** 1 Health Promotion and System Strengthening/Tuimarishe Afya Project, 7th Road, ACT Building, P.O. Box 29, Dodoma, Tanzania; 2 Swiss Centre for International Health at Swiss Tropical and Public Health Institute, Socinstrasse 57, P.O. Box 4002, Basel, Switzerland; 3 University of Basel, P.O. Box, CH-4003, Basel, Switzerland; 4 Dodoma Regional Medical Officer’s Office, P.O. Box 914, Dodoma, Tanzania; 5 Department of Microbiology and Immunology, Weill Bugando School of Medicine, Catholic University of Health and Allied Sciences, P. O. Box 1464, Mwanza, Bugando Tanzania

**Keywords:** Hand hygiene, Health care facilities, Tanzania

## Abstract

**Background:**

Compliance with guidelines on hand hygiene (HH) is pivotal to prevent and control health-care associated infections and contributes to mitigating antimicrobial resistance. A baseline assessment in Dodoma region, Tanzania in March 2018 showed inadequate HH levels across health care facilities. We evaluated the impact of training in HH as part of a water, sanitation and hygiene (WASH) interventions of “Maji kwa Afya ya Jamii” (MKAJI) project.

**Methods:**

A comparative HH assessment was conducted in June 2019 involving health care facilities under MKAJI project (*n* = 87 from which 98 units were assessed) vs non-MKAJI facilities (*n* = 85 from which 99 units were assessed). Irrespective of MKAJI interventional status, baseline assessment in March 2018 were compared to re-assessment in June 2019 in all health care facility units (unpaired comparison: 261 vs 236 units, respectively), and in facilities assessed in both surveys (paired comparison: 191 versus 191 units, respectively). The ‘WHO HH Self-Assessment Framework Tool, 2010’ with five indicators each counting 100 points was used. The cumulative scores stratified each health facility’s unit into inadequate (0–125), basic (126–250), intermediate (251–375) or advanced (376–500) HH level (score). The HH compliance rates were also assessed and compared.

**Results:**

The overall post-intervention median HH score [interquartile range (IQR)] was 187.5 (112.5–260). MKAJI health facilities had significantly higher median HH scores (IQR) [190 (120–262.5)] compared with non-MKAJI facilities [165 (95–230); *p* = 0.038]. Similarly, the HH compliance rate of ≥51% was significantly higher in MKAJI than non-MKAJI facilities [56.1% versus 30.3%; chi2 = 13.39, *p* < 0.001]. However, the recommended WHO compliance rate of ≥81% was only reached by 6.1 and 3.0% units of MKAJI and non-MKAJI facilities, respectively. Both paired and unpaired comparisons during baseline and re-assessment surveys showed increase in HH level from inadequate to basic level.

**Conclusion:**

The overall HH level after the combined WASH and training intervention was at basic level. Higher median HH scores (IQR) and HH compliance rates were evident in health facilities of the MKAJI project, underscoring the impact of the intervention and the potential value of a national roll-out.

## Introduction

Healthcare-associated infections (HCAIs) are challenging health care facilities across the world [1]. The burden of HCAIs is further complicated by a particularly high prevalence of multi-drug resistant (MDR) pathogens in hospitals, resulting in significant morbidity, mortality and extra health-care expenditure [2–4]. In the United Republic of Tanzania (Tanzania hereinafter), MDR infections are higher among patients admitted in hospitals than those with community-associated infections. Reports from Mwanza city and Dodoma – Tanzania’s capital city showed that the proportions of women developing surgical site infections post-caesarean section were 11 and 48%, respectively [5, 6]. Moreover, children under 5 years of age, specifically neonates with sepsis remain also vulnerable to MDR infections [7, 8].

The clonal spread of these pathogens suggests a common source [9–11]; however, delineation of the ultimate source remains to be explored. Various reports on infection prevention and control (IPC) in Tanzania have stipulated specific guidelines, standard procedures and communication strategies to ensures IPC and ultimate patient safety [12–14]. Nevertheless, there are a number of challenges to address and overcome, including scarcity of material resources/items required for IPC, a lack of technical know-how by medical personnel, administrative, logistical and financial constraints [12–15].

Despite the fact that adherence to hand hygiene practices was shown to be pivotal in reducing carriage of MDR pathogens by healthcare workers’ hands and subsequent transmission to patients, compliance has plateaued at around 40% in a multi-centre studies across the world [16–18]. To ensure uniformity and objective assessment of hand hygiene practices, the ‘WHO Hand Hygiene Self-Assessment Framework Tool (2010)’ was introduced to promote hand hygiene [17, 19]. This multimodal strategy has been designed to take into account individuals as well as system related factors in ascertaining the healthcare workers compliance with the IPC measures when providing routine care to the patients in health facilities, irrespective of the level of health facilities or economic status [17].

An extensive global assessment on water, hygiene and sanitation (WASH) conducted jointly by the WHO and UNICEF in 2016 showed that globally approximately three quarters of health care facilities have basic water services, with 55% availability in least developed countries. One out of six health care facilities (16%) worldwide is estimated to have no hygiene services at all, translating into 896 million people with no access to hygiene services in their health care facilities. Of note, availability of water services was estimated to be three times less in the rural health care facilities compared with those in urban settings [20]. This situation poses a wide-reaching challenge to the Sustainable Development Goals (SDGs) outlined in the Agenda 2030 on Sustainable Development – above all – SDG 3 (‘to ensure healthy lives and promote well-being for all at all ages’) and SDG 6 (‘to ensure availability and sustainable management of water and sanitation for all’) [21].

In April 2017, Tanzania launched its National Action Plan on AMR (2017–2022) in response to the global action plan on combating AMR [22, 23]. In this national plan, the Priority Area 6 is focusing on IPC in health care systems with hand hygiene being a critical component [22]. In this context, health centres and dispenasries in Dodoma region received support for WASH interventions by the ‘Maji kwa Afya ya Jamii’ (MKAJI) project aiming to upgrade water supply and sanitation in these primary health facilities (https://www.eda.admin.ch/dam/countries/countries-content/tanzania/en/601.0-00_Factsheet _SDC_MKAJI_EN.pdf). The MKAJI project covered a total of 90 health care facilities between 2014/15 and 2018/19; with activities ongoing in four additional health care facilities. WASH infrastructure and capacity development/training among health care workers was provided by the project. A baseline assessment on hand hygiene among health care facilities in Dodoma region in March 2018 demonstrated inadequate levels of hand hygiene calling for a more refined strategy to address this low compliance, notably in dispensaries and health centres (Wiedenmayer & Seni., 2018, unpublished data available at http://hssrc.tamisemi.go.tz/storage/app/uploads/public/5bf/a92/34c/5bfa9234cb9f1296356496.pdf).

To explore whether the capacity building and WASH training provided within MKAJI had an effect on hand hygiene, the Swiss-funded Health Promotion and System Strengthening (HPSS) project planned to conduct a comparative study between those health care facilities under the MKAJI interventional project and non-MKAJI facilities to guide future IPC measures. Moreover, a comparison between the baseline level of hand hygiene obtained in March 2018 and the re-assessment in June 2019 post-intervention was conducted.

## Methodology

### Assessment design and settings

This was a comparative study which involved MKAJI and non-MKAJI health care facilities in Dodoma region in June 2019. Dodoma region hosts the capital city of Tanzania and is located in the central zone of the country. It has a population of 2,083,588 as per National Housing and Population Census, 2012. Dodoma region is divided into 8 district councils (DC) with 8 hospitals, 30 health centres and 284 dispensaries. The district councils are Dodoma City Council, Chamwino DC, Kondoa DC, Kondoa Town Council, Bahi DC, Chemba DC, Mpwapwa DC and Kongwa DC. However, in the analysis Kondoa DC and Kondoa Town Council were combined together.

### Study population, sampling strategy and sample size

A total of 236 participants (out of 242 eligible participants) from 7 hospitals, 16 health centres and 155 dispensaries were recruited in June 2019. Of these, 87 were MKAJI health care facilities (seven health centres and 80 dispensaries from which 98 units were assessed) and 85 non-MKAJI health care facilities (9 health centres and 75 dispensaries from which 99 units were assessed)*.* One unit was included for assessment for dispensaries (i.e. labour wards/rooms), 3 units were included in health centres (i.e. labour wards/rooms, theatre and outpatient) and 6 units were included in hospitals (i.e. labour ward, theatre, outpatient, laboratory, pharmacy and surgical wards). Individuals who were in-charge of a health facility’s unit and who gave their consent to be involved in the study were included (i.e. all 236 participants from 236 units were included)*.* Health care facility units under MKAJI were compared to units not under MKAJI interventional project based on the hand hygiene scores, levels and compliance. Furthermore and irrespective of MKAJI interventional status, baseline hand hygiene scores and their corresponding levels (obtained in March 2018) were compared with those obtained in this post-interventional re-assessment i.e. in June 2019. In the later, unpaired comparison included all health care facility units assessed (261 units in March 2018 versus 236 units in June 2019), and paired comparison involved only health care facility units which were both involved in the baseline and re-assessment (191 versus 191 units, respectively).

This project was approved by institutional board [the Joint Catholic University of Health and Allied Sciences/Bugando Medical Centre Research and Ethics Committee (CREC/358/2019)] and the National Institute for Medical Research (NIMR/HQ/R.8a/Vol. IX/3116).

### Data management

#### Sources of data

Data were collected from in-charges of health facility’s units via interviews and observations using the ‘WHO Hand Hygiene Self-Assessment Framework 2010 Tool’. This tool is divided into five components containing 27 indicators. The five components are i) system change (SC); ii) training and education (TE); iii) evaluation and feedback (EF); iv) reminders in the work place (RW); and v) institutional safety climate (ISC). Each of these components has a subtotal score of 100, amounting to an overall maximum hand hygiene score of 500 [19].

#### Preparation for data collection

The project assessment team underwent a 5-day training session on the general principles of hand hygiene in the context of IPC, the assessment protocol and pre-testing of the data collection tool in six dispensaries in Dodoma city council. This was followed up by a feedback session, where questions, inquiries and concerns were addressed ensuring that all research assistants would be conversant with the data collection tool. The same research assistants deployed in the baseline assessment in March 2018, were involved in the re-assessment in June 2019.

#### Data quality checks

Research assistants were divided into three groups each with a team lead. The later was tasked to oversee the data quality at the end of each day, and do the necessary corrective actions. Then, filled data collection tools were sent to the data quality officer on weekly basis. The final data quality assessment was done by the co-investigators and the principal investigator.

#### Data analysis

Data collected was entered into an Excel sheet for cleaning and consistency checks and then exported to STATA version 13.0 software (StataCorp®, College Station, Texas, USA) for analysis. Cumulative scores of five indicators stratified each health facility’s unit into inadequate (0–125), basic (126–250), intermediate (251–375) or advanced (376–500) hand hygiene (score) level. Categorical variables such as type of professional cadre, health care facilities levels (i.e. dispensary, health centre or hospital) and hand hygiene levels were described as proportions and compared using a Chi-squared (Chi2) test. Participants’ ages were presented as mean ± standard deviation, whereas the hand hygiene scores were presented by median scores (interquartile range). Comparison of median hand hygiene scores in various variables such as district councils, health facility ranks, health facility units and MKAJI project status was done using a two-sample Wilcoxon rank-sum (Mann-Whitney) test. The significance cut off was set at *p* < 0.05 for associations between hand hygiene level/score and other variables.

## Results

### Demographic information of participants and health care facilities

The majority of participants were female (60.2%) and 112 (47.5%) were nurses. Other professional cadres were clinicians 23.7% (medical specialists, medical doctors, assistant medical doctors, clinical officers and clinical assistants), medical attendants 19.1%, laboratory staff 5.5%, pharmacy staff 2.5%, and others 1.7%. The mean age ± SD of participants was 34.4 ± 9.8 years, ranging from 20 years to 60 years.

The overall distribution of the 236 health facility units across seven district councils was similar (range: 27 to 33 per council), with exception of Dodoma city council which accounted for 20.7% of all units. The majority of units involved were labour wards/rooms (75.0%) followed by outpatient units (10.2%) (Table 1).

**Table 1:** Post-interventional hand hygiene scores and levels by health facility units in Dodoma region

Health facility unit	Median hand hygiene score (IQR)	Hand hygiene level
Labour ward (*n* = 177)	182.5 (105–250)	Basic
Outpatient (*n* = 24)	205 (143.8–272.5)	Basic
Theatre (*n* = 14)	185 (115–250)	Basic
Laboratory (n = 8)	197.5 (111.3–307.5)	Basic
Pharmacy (n = 7)	180 (115–195)	Basic
Surgical ward (n = 6)	327.5 (235–347.5)	Intermediate
Total (*N* = 236)		

*IQR* Interquartile range

### Hand hygiene scores and levels across health care facilities in Dodoma region

#### Hand hygiene scores and levels by health care facility units and district councils

The overall post-intervention median hand hygiene score (IQR) across 236 health care facility units in Dodoma region was 187.5 (112.5–260), with the minimum and maximum scores being 25 and 425, respectively. The distribution of hand hygiene levels across health care facility units post-intervention were inadequate 31.4% (*n* = 74), basic 40.3% (*n* = 95), intermediate 25.4% (*n* = 60), and advanced 3.0% (n = 7). The median hand hygiene score (IQR) was significantly higher in hospital units [235 (147.5–320) than in dispensaries [175 (100–247.5); *p* = 0.0136]. Although not statistically significant, median hand hygiene score (IQR) was also higher in health centre units [188.8 (115–260)] than in dispensaries [175 (100–247.5); *p* = 0.458]. All health care facility units had basic hand hygiene levels, except surgical units which had an intermediate hand hygiene level (Table 1). All seven city/district councils in Dodoma region had basic hand hygiene level, except Bahi district council which had inadequate hand hygiene level with median hand hygiene score (IQR) of [90 (70–130)].

#### Evaluation of hand hygiene indicators in health facility units

Post-intervention evaluation of the five hand hygiene indicators in 236 units across various health care facilities in Dodoma region showed that the respective median hand hygiene score (IQR) were SC [45 (35–55)] and EF [50 (25–67.5). These indicators were relatively higher compared to TE [25 (5–55)], RW [30 (5–50)] and ISC [37.5 (20–60)]. Continuous supply of clean and running water was observed in approximately 90.3% (213/236), whereas the presence of alcohol hand rub was observed in 30.9% (73/236) of the health care facility units. It was reported that TE among health care workers on hand hygiene had never been received in approximately 44.1% (*n* = 104), whereas in 37.7% (*n* = 89), training has been received only once. On the other hand, approximately 14.8% (*n* = 35) and 3.0% (*n* = 8) have regular and mandatory training in their workplaces, respectively.

**Fig. 1:**
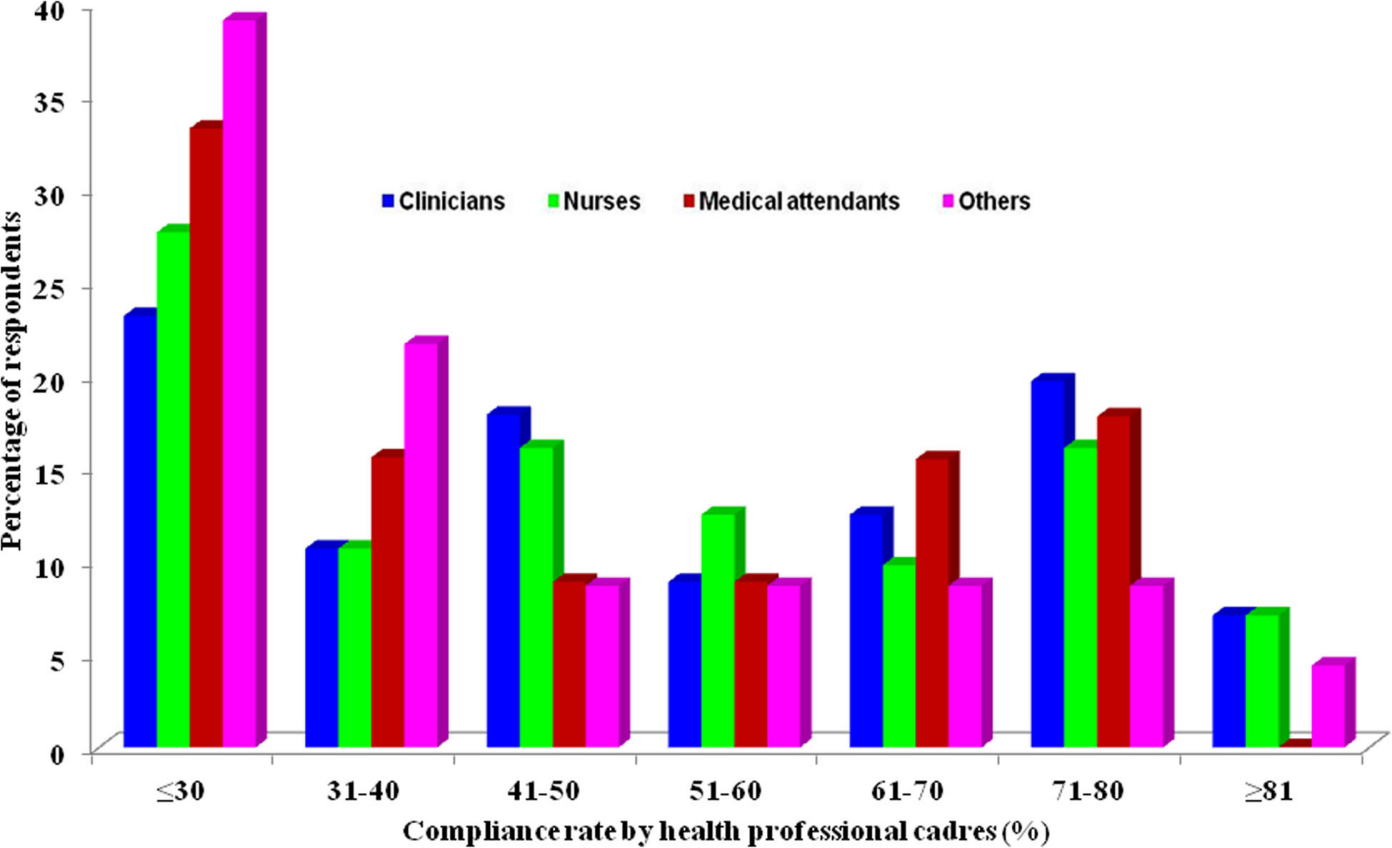
Self-reported hand hygiene compliance using WHO tool (or similar techniques) among health workers by professional cadres

The compliance rates were relatively higher among clinicians and nurses, compared to medical attendants and other professional cadres (Fig. 1). The compliance rates above 50% and the WHO recommended rate of ≥81.0% specific to each professional cadres were found to be as follows; clinicians 48.2% (*n* = 27) and 7.2% (*n* = 7), nurses 45.5% (*n* = 51) and 7.1% (*n* = 8), medical attendants 42.2% (*n* = 19) and 0.0%(*n* = 0) and others 30.5% (*n* = 7) and 4.4% (*n* = 1), respectively (Fig. 1).

### Comparison of hand hygiene scores between MKAJI health care facilities and non-MKAJI facilities

**Fig. 2:**
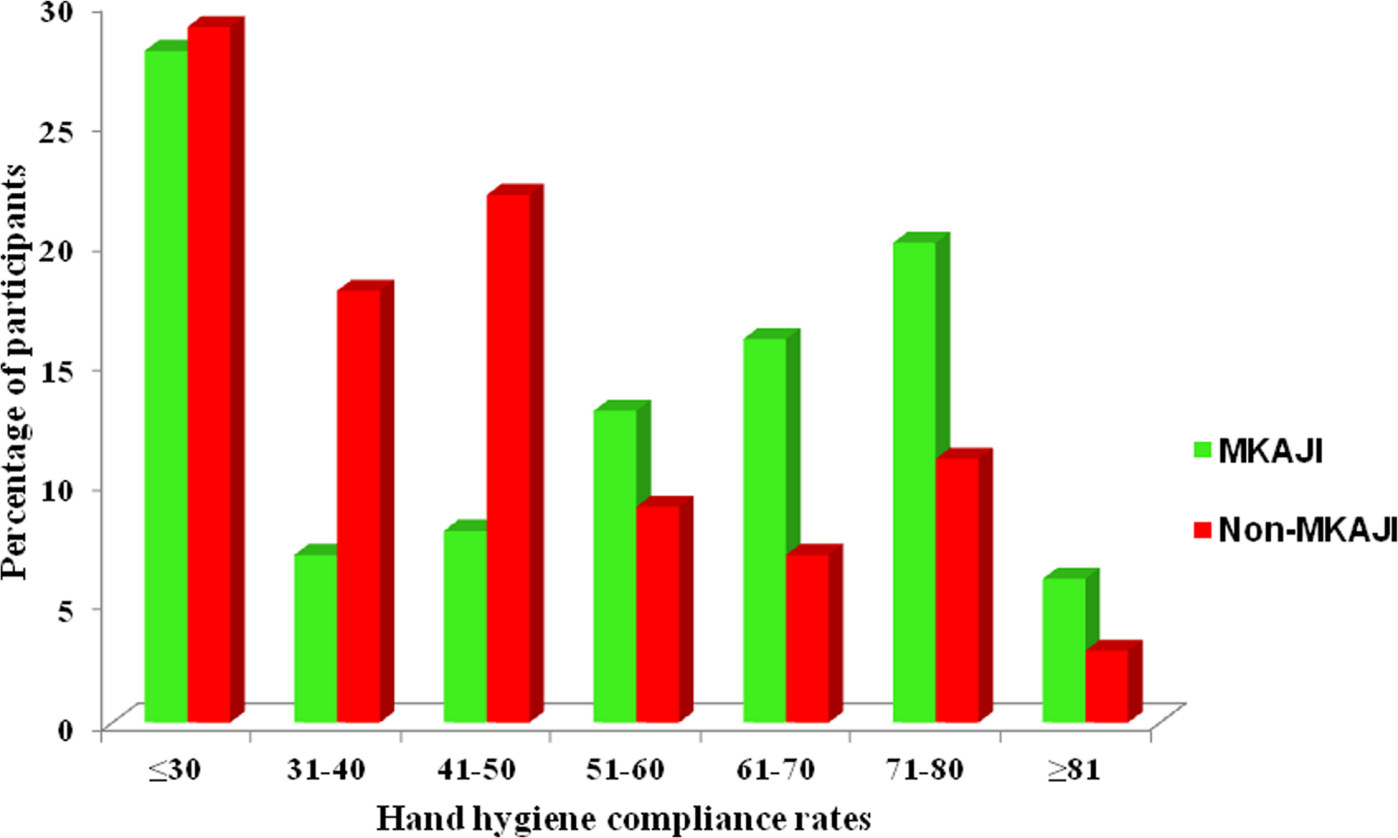
Comparison of self-reported hand hygiene compliance using WHO tool (or similar techniques) among health workers by MKAJI status

Of the 236 health care facility units, 197 units from health centres and dispensaries were subjected to a sub-analysis. Those health care facility units involved in the MKAJI intervention project had significantly higher median hand hygiene scores (IQR) [190 (120–262.5)] compared with non-MKAJI facilities [165 (95–230); *p* = 0.038]. The overall median hand hygiene scores (IQR) were also higher among MKAJI health care facilities compared with non-MKAJI facilities in both labour wards [190 (120–265) versus 166.3 (90–230)]; and outpatient units [178.8 (155–243.8) versus 147.5 (127.5–260)]. The median hand hygiene scores (IQR) differences between the two groups were also evident in all district councils. The hand hygiene scores (IQR) for indicators which demonstrated significant differences between MKAJI versus non-MKAJI units were TE [32.5 (5–55) versus 10 (0–40), *p* < 0.001); and EF [55 (25–70) versus 40 (20–60), *p* = 0.043]. On the other hand, the remaining three indicators did not show significant differences: SC [45 (35–50) versus 40 (30–50), *p* = 0.288]; RW [22.5 (5–50) versus 26.3 (5–47.5), *p* = 0.877), and ISC [35 (20–60) versus 35 (15–55), *p* = 0.408)]. There was no statistical difference on the availability of continuous supply of water in units associated with MKAJI versus non-MKAJI units [90.8% (89/98) versus 88.9% (88/99); chi2 = 0.201; *p* = 0.654]. Similarly, no statistical difference was observed on the availability of alcohol hand rub in MKAJI units [29.6% (29/98) versus 23.2% (23/99); chi2 = 1.025; *p* = 0.311]. Hand hygiene compliance rate of ≥51% was significantly higher in MKAJI units than non-MKAJI units [56.1% (55/98) versus 30.3% (30/99); chi2 = 13.39; *p* < 0.001]. However, the recommended WHO compliance rate of ≥81% was only reported in 6.1 and 3.0% for MKAJI and non-MKAJI units, respectively (Fig. 2).

### Baseline hand hygiene versus hand hygiene re-assessment in Dodoma region irrespective of MKAJI intervention status

#### Overall baseline and re-assessment hand hygiene score across health care facilities

While the overall hand hygiene level among the 261 units in Dodoma region was found to be inadequate during the baseline [median score (IQR): 80 (60–145], it increased to basic level after the re-assessment in 2019 [median score (IQR): 187.5 (112.5–260; *p* < 0.001] (Table 2).

**Table 2:** Comparison of baseline and hand hygiene re-assessment in health care facility units in Dodoma region

Health care facilities	Baseline median HH score (IQR) (*n* = 261 units)	Re-assessment median HH score (IQR) (*n* = 236 units)
Hospitals	107 (80–182.5)	235 (147.5–320)
Health centres	76.3 (60–125)	188.8 (115–260)
Dispensaries	75 (55–145)	175 (100–247.5)
Overall median HH score (IQR)	80 (60–145	187.5 (112.5–260

#### Paired comparison of hand hygiene among facilities sampled at the baseline and during re-assessment in Dodoma region

**Fig. 3:**
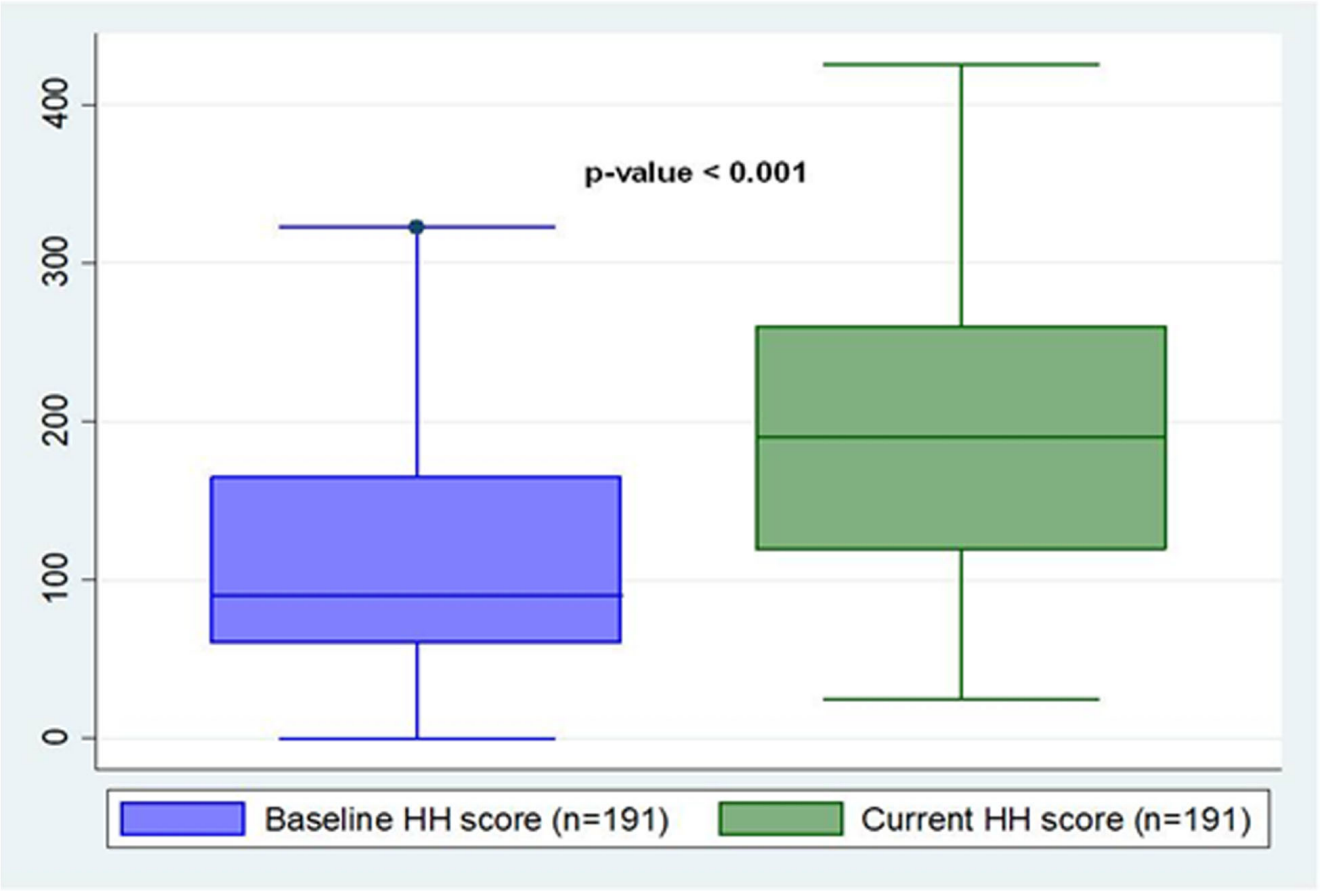
Paired comparison of baseline and follow-up hand hygiene re-assessment in Dodoma region

Of the 236 health care facility units involved in the current study, only 191 (80.9%) were enrolled in the baseline assessment in March 2018 and therefore, the later allowed for a specific sub-analysis to assess a change in trend. While the overall hand hygiene level was inadequate at baseline [IQR of 90 (60–165)], the level of those same 191 units had increased to basic level by the time of the re-assessment [IQR of 190 (120–260); (*p* < 0.001)] (Fig. 3).

## Discussion

Approximately 90% of the health care facilities involved in this study were dispensaries, whereas the remaining facilities being either health centres or hospitals, and the majority (75.0%) of units were labor wards. This higher proportion of labor wards is because this unit is present in all three levels of health care facilities i.e. dispensaries, heath centres and hospitals. Previous studies in various units in Ethiopia, China and Switzerland have reiterated the need for a wide coverage of units to allow generalization of the findings in the context of patients care services [24–26]. Similar to previous work in Ethiopia, China and Italy, the current study showed a predominance of females and nurses [24, 25, 27]. This may be related to the fact that the nursing profession is a predominant health cadre in Tanzania irrespective of the rank of the health care facilities.

The overall post-intervention hand hygiene level was basic with a median score of 187.5, and was higher than the inadequate level reported in the baseline assessment in March 2018 (both in paired and unpaired comparisons). This is comparable to a similar study in India which reported a hand hygiene score of 225 [28]. Similar to the baseline assessment, the hand hygiene score was higher in hospitals than in health centres and dispensaries. The relatively higher level of performance in hospitals as opposed to health centres and dispensaries may relate to the fact that most hospitals are better equipped with material resources, have higher staff numbers and better access to information regarding hand hygiene. The significant improvement since the baseline assessment may be attributed to the on-going MKAJI project interventional measures, post-baseline assessment sensitization sessions and provision of hand hygiene tools to various health care facilities by the HPSS project and in collaboration with the council health management teams. In this regard, strengthening of hand hygiene activities in health centres and dispensaries should be emphasized so as to have comprehensive coverage and subsequently reduce potential HCAIs in all ranks/tiers of health care facilities in Dodoma region and other areas in Tanzania with similar epidemiological predisposition [1, 16].

It was evident that continuous supply of clean water through conventional or improvised sinks was remarkably high (90.3%), in contrast to the presence of alcohol-based hand rub which was observed in only one third of the health care facilities’ units. Nevertheless, our finding on alcohol based hand rub was higher than the 11.5% reported from a previous study in Ethiopia [24]. The causes of low utilization of alcohol based hand rub in Tanzania and Ethiopia were not evaluated; however, studies in Kenya and China demonstrated that smell, skin irritation, dryness, unreliable availability, and heavy work load negatively affected hand hygiene practices among health workers [25, 29]. In contrast to these factors, in a busy hospital setting like an intensive care unit, alcohol based hand rub is preferred due to its convenience [30]. Therefore, assessment of specific individual, institutional and government factors affecting hand hygiene will be of interest in the future assessments in Dodoma region.

Regarding the self-reported hand hygiene compliance, good compliance was reported more frequently among clinicians and nurses, as opposed to other professional cadres. Of note, none of the medical attendants had achieved the WHO recommended rate of ≥81.0%. The compliance rate in this study was low compared to the baseline compliance rates reported in Kenya (28%), Ethiopia (22.0%), China (66.3%), Switzerland (61.4%) and in a systematic review involving 96 studies (40%) [18, 24–26]. However, variable compliance has been previously reported in six ICU in Italy ranging from 3 to 100% [27]. Variability in the hand hygiene compliance rates across countries may be related to individual, institutional and government differences with regard to hand hygiene practices, resource availability and reinforcement modalities available in each setting. Interestingly, in other countries where a baseline assessment was done followed by specific hand hygiene interventions, the re-assessment showed remarkably increased hand hygiene compliance rate irrespective of the health workers’ profession and hospital units [18, 24–26, 29]. Although the hand hygiene compliance rate of ≥81% recommended by WHO was reported to be significantly lower in both types of health care facilities assessed (MKAJI and non-MKAJI associated), the overall compliance rate was higher among respondents from MKAJI units. Low compliance was also reported in Kenya (28% pre-intervention to 38% post-intervention, respectively) as opposed to higher compliance (48–88%) in China connoting similar epidemiological and infrastructural predisposition in Tanzania and Kenya [24, 25, 29]. These findings emphasize that specific interventions, when carefully designed, can have a significant positive impact, which in turn can improve patients’ health care services. Therefore, similar programs should be rolled out to increase coverage not only in Dodoma but also in other regions in Tanzania.

This assessment did not evaluate all health facilities in Dodoma region. Nevertheless, over three quarters of health care facilities were assessed allowing for extrapolation of the findings to the rest of the facilities in this region. The hand hygiene compliance was self reported and subjectivity elements cannot be ruled out. However, audit files were used for verification purposes. This study could not link the impacts of hand hygiene levels/scores to AMR rates and patients’ outcomes, and these parameters should be studied in future investigations.

## Conclusions

The overall hand hygiene level in healthcare facilities in Dodoma region increased from inadequate level in March 2018 to basic level in June 2019. Hand hygiene practice was significantly higher in hospitals compared with health centres and dispensaries. There was significantly higher hand hygiene score and hand hygiene compliance rate in health care facilities associated with the MKAJI interventional project. However, the recommended WHO compliance rate of ≥81% was widely missed by both MKAJI and non-MKAJI units. Most health facilities had a continuous supply of water but only one third of facilities provided alcohol-based hand rub.

Future assessments should maintain regular region-wide hand hygiene evaluation. Programs similar to MKAJI should be rolled out to increase the coverage not only in Dodoma but also in other regions in Tanzania. Ascertaining the implication of the hand hygiene scores/levels and its compliance in relation to the incidence of HCAIs and AMR rates would be of interest in future studies in order to translate these figures directly into patients’ care and health systems’ performance.

## Data Availability

The datasets used and/or analysed during the current study are available and can be accessed from the corresponding author on reasonable request.

## Abbreviations

AMR: Antimicrobial resistance
EF: Evaluation and feedback
HCAIs: Health care associated infections
IPC: Infection prevention and control
IQR: Interquartile range
ISC: Institutional safety climate
MDR: Multidrug resistance
MKAJI: “Maji kwa Afya ya Jamii” (a Swahili phrase: “Water for Healthier Community”)
RW: Reminders in the work place
SC: System change
TE: Training and education
WASH: Water, hygiene and sanitation
WHO: World Health Organization

## References

[1] Nejad SB, Allegranzi B, Syed SB, Ellis B, Pittet D (2011). Health-care-associated infection in Africa: a systematic review. Bull World Health Organ.

[2] Peleg AY, Hooper DC (2010). Hospital-acquired infections due to gram-negative bacteria. N Engl J Med.

[3] WHO: Antimicrobial resistance: global report on surveillance: World Health Organization; 2014.

[4] Seni J, Moremi N, Matee M, van der Meer F, DeVinney R, Mshana SE, JD DP: Preliminary insights into the occurrence of similar clones of extended-spectrum beta-lactamase-producing bacteria in humans, animals and the environment in Tanzania: a systematic review and meta-analysis between 2005 and 2016. Zoonoses Public Health 2018 Feb*;*65*(*1*):*1*–*10*. doi:* 10.1111/zph.12387*.*. 10.1111/zph.12387 28834351

[5] Mpogoro FJ, Mshana SE, Mirambo MM, Kidenya BR, Gumodoka B, Imirzalioglu C (2014). Incidence and predictors of surgical site infections following caesarean sections at Bugando medical Centre, Mwanza, Tanzania. Antimicrob Resist Infect Control.

[6] De Nardo P, Gentilotti E, Nguhuni B, Vairo F, Chaula Z, Nicastri E, Nassoro MM, Bevilacqua N, Ismail A, Savoldi A (2016). Post-caesarean section surgical site infections at a Tanzanian tertiary hospital: a prospective observational study. J Hosp Infect.

[7] Blomberg B, Manji KP, Urassa WK, Tamim BS, Mwakagile DS, Jureen R, Msangi V, Tellevik MG, Holberg-Petersen M, Harthug S (2007). Antimicrobial resistance predicts death in Tanzanian children with bloodstream infections: a prospective cohort study. BMC Infect Dis.

[8] Seni J, Mwakyoma AA, Mashuda F, Marando R, Ahmed M, DeVinney R, Pitout JDD, Mshana SE (2019). Deciphering risk factors for blood stream infections, bacteria species and antimicrobial resistance profiles among children under five years of age in North-Western Tanzania: a multicentre study in a cascade of referral health care system. BMC Pediatr.

[9] Moremi N, Mshana SE, Kamugisha E, Kataraihya J, Tappe D, Vogel U, Lyamuya EF, Claus H (2012). Predominance of methicillin resistant Staphylococcus aureus -ST88 and new ST1797 causing wound infection and abscesses. J Infect Dev Countries.

[10] Mshana SE, Hain T, Domann E, Lyamuya EF, Chakraborty T, Imirzalioglu C (2013). Predominance of Klebsiella pneumoniae ST14 carrying CTX-M-15 causing neonatal sepsis in Tanzania. BMC Infect Dis.

[11] Marando R, Seni J, Mirambo MM, Falgenhauer L, Moremi N, Mushi MF, Kayange N, Manyama F, Imirzalioglu C, Chakraborty T (2018). Predictors of the extended-spectrum-beta lactamases producing Enterobacteriaceae neonatal sepsis at a tertiary hospital, Tanzania. Int J Med Microbiol.

[12] JHPIEGO.: The United Republic of Tanzania Ministry of Health and Social Welfare National Infection Prevention and Control Standards for Hospitals in Tanzania Standards- Standards-Based Management and Recognition for Improving Infection Prevention And Control Practices – An Assessment Tool June 2012. 2012.

[13] URT: United Republic of Tanzania. National Communication Strategy for Infection Prevention and Control 2012-2017. . 2012.

[14] URT: United Republic of Tanzania. National Infection Control Guidelines. Draft for consultation. 2016.

[15] Jones M, Gower S, Whitfield A, Thomas S (2015). Evaluation of practice change in Tanzanian health professionals 12 months after participation in an infection prevention and management course. J Infect Prev.

[16] Allegranzi B, Pittet D (2009). Role of hand hygiene in healthcare-associated infection prevention. J Hosp Infect.

[17] WHO: A guide to the implementation of the WHO Multimodal Hand Hygiene Improvement Strategy. who/iep/psp/2009.2. 2009.

[18] Erasmus V, Daha TJ, Brug H, Richardus JH, Behrendt MD, Vos MC, van Beeck EF (2010). Systematic review of studies on compliance with hand hygiene guidelines in hospital care. Infect Control Hosp Epidemiol.

[19] WHO: WHO guidelines on hand hygiene in health care. First global patient safety challenge. Clean care is safer care. 2009. 23805438

[20] WHO & UNICEF: WASH in health care facilities: global baseline report 2019. Geneva: World Health Organization and the United Nations Children’s Fund (UNICEF), 2019. Licence: CC BY-NC-SA 3.0 IGO. 2019.

[21] UNDP: United Nations Development Programme. Sustainable Development Goals. Available at http://www.un.org/sustainabledevelopment/news/communications-material/. New York, NY 10017 USA. 2016.

[22] URT: The National Action Plan on Antimicrobial Resistance (2017–2022). The Ministry of Health Community Development Gender Elderly and Children (MHCDGEC), Ministry of Agriculture, Livestock and Fisheries (MALF). The United Republic of Tanzania (URT). 2017.

[23] WHO: Global Action Plan on Antimicrobial Resistance. The World Health Organization, Geneva, Switzerland. (http://apps.who.int/gb/ebwha/pdf_files/ WHA68/A68_20-en.pdf?ua=1). 2015.

[24] Kolola T, Gezahegn T (2017). A twenty-four-hour observational study of hand hygiene compliance among health-care workers in Debre Berhan referral hospital, Ethiopia. Antimicrob Resist Infect Control.

[25] Shen L, Wang X, An J, An J, Zhou N, Sun L, Chen H, Feng L, Han J, Liu X (2017). Implementation of WHO multimodal strategy for improvement of hand hygiene: a quasi-experimental study in a traditional Chinese medicine hospital in Xi’an, China. Antimicrob Resist Infect Control.

[26] Staines A, Vanderavero P, Duvillard B, Deriaz P, Erard P, Kundig F, Juillet C, Clerc O. Sustained improvement in hand hygiene compliance using a multimodal improvement program at a Swiss multisite regional hospital. J Hosp Infect. 2018;100(2):176–82. 10.1016/j.jhin.2018.04.010 29654810

[27] Musu M, Lai A, Mereu NM, Galletta M, Campagna M, Tidore M, Piazza MF, Spada L, Massidda MV, Colombo S (2017). Assessing hand hygiene compliance among healthcare workers in six intensive care units. J Prev Med Hyg.

[28] Sharma R, Sharma M, Koushal V (2014). Compliance to hand hygiene world health organization guidelines in hospital care. Int J Prev Med.

[29] Ndegwa L, Hatfield KM, Sinkowitz-Cochran R, D'Iorio E, Gupta N, Kimotho J, Woodard T, Chaves SS, Ellingson K (2019). Evaluation of a program to improve hand hygiene in Kenyan hospitals through production and promotion of alcohol-based Handrub - 2012-2014. Antimicrob Resist Infect Control.

[30] Saito H, Kilpatrick C, Pittet D (2018). The 2018 World Health Organization SAVE LIVES: clean your hands campaign targets sepsis in health care. Intensive Care Med.

